# Impact of COVID-19 on Ventilation-Perfusion (V/Q) Scans: A Comparative Analysis of Pre-COVID-19 Era V/Q Scans and Post-COVID-19 Era Perfusion Scans

**DOI:** 10.7759/cureus.66434

**Published:** 2024-08-08

**Authors:** Pokhraj P Suthar, Karl Villanueva, Sumeet Virmani

**Affiliations:** 1 Department of Diagnostic Radiology and Nuclear Medicine, RUSH University Medical Center, Chicago, USA

**Keywords:** hypercoagulability, pulmonary embolism, lung perfusion scan, ventilation-perfusion scan, covid-19

## Abstract

Introduction

The COVID-19 pandemic has profoundly impacted medical practices, including nuclear medicine. To minimize aerosol transmission risks, lung perfusion scintigraphy was preferred over traditional ventilation-perfusion (V/Q) scintigraphy during the pandemic. This study compares lung perfusion scans performed during COVID-19 with V/Q scans from the pre-COVID era. After reviewing this study, the reader will learn about V/Q scintigraphy and lung perfusion.

Methods

This retrospective observational study, conducted from December 2018 to July 2021, involved 868 patients - 511 in the pre-COVID era and 357 in the post-COVID era - at a single tertiary care center. The pretest probability of pulmonary embolism (PE) was determined using Wells' criteria, and data including demographics, clinical findings, and diagnostic test results (V/Q or lung perfusion scintigraphy) were collected.

Results

A 30% decline in lung scans was observed during the pandemic. In the pre-COVID era, 68.3% of scans had low, 27.8% had intermediate, and 3.9% had high probability for PE. During the pandemic, perfusion-only scans showed 57.3% low, 32.9% indeterminate, and 9.8% high probability for PE. Among COVID-19-positive patients, 48.9% had intermediate, and 11.1% had high probability scans. The rise in indeterminate and high-probability scans during the pandemic is attributed to COVID-19-related lung changes and hypercoagulability.

Conclusion

The perfusion component of lung scans is typically sufficient for evaluating acute PE. Omitting the ventilation part of the V/Q scan had minimal impact, with only a 5.1% increase in indeterminate/non-diagnostic scans using perfusion-only modified Prospective Investigation of Pulmonary Embolism Diagnosis II (PIOPED II) criteria during the post-COVID-19 era, likely due to underlying lung parenchymal involvement in COVID-19 patients. Additionally, there was a 5.9% rise in high-probability scans, attributed to the hypercoagulability and vascular complications associated with COVID-19.

## Introduction

The worldwide rapid emergence of the COVID-19 pandemic has significantly altered medical practices. In the United States alone, approximately 103 million documented COVID-19 cases, and about 1.2 million deaths have been reported by June 2024 [1]. COVID-19 is caused by the single-strand RNA virus SARS-CoV-2, which spreads through aerosols. To prevent contamination and the spread of COVID-19, nuclear medicine modified ventilation-perfusion (V/Q) scintigraphy to lung perfusion scintigraphy only. This change eliminated the ventilation portion of the study, thereby minimizing the potential for aerosolization and patient-to-patient cross-contamination. Lung V/Q scintigraphy, first used in humans in 1964, is the oldest noninvasive diagnostic study for evaluating pulmonary embolism (PE). Until computed tomography (CT) was introduced in 1978, V/Q scans were the sole imaging modality for PE [2,3]. COVID-19, transmitted through respiratory droplets, can remain viable on surfaces such as plastic and stainless steel for up to three days. The U.S. Centers for Disease Control and Prevention (CDC) recommends healthcare personnel wear N95 respirators, gloves, gowns, and eye protection during aerosol-generating procedures to minimize infection risk [4-6]. COVID-19 increases the risk of PE due to factors such as hypercoagulability, endothelial damage, prolonged immobilization, and severe inflammatory responses. The ventilation phase of the V/Q scan carries a risk of radioactive contamination due to aerosol leakage, especially during the COVID-19 pandemic. Consequently, many institutions, including ours, adopted perfusion-only studies to mitigate this risk, following guidelines from the Society of Nuclear Medicine and Molecular Imaging (SNMMI). Despite a global reduction in lung scans during the pandemic, our study observed increased intermediate and high-probability scans, likely due to lung parenchymal changes and higher rates of thrombotic complications in COVID-19 patients. Lung perfusion scintigraphy combined with chest radiography provides diagnostic accuracy comparable to V/Q scans and computed tomography pulmonary angiography (CTPA) but at a lower radiation dose and cost. While single-photon emission computed tomography (SPECT)/CT imaging offers higher accuracy, it comes with additional radiation exposure. This study provides a comparative analysis of lung perfusion scans performed during the COVID-19 pandemic to V/Q scans performed during the immediate pre-COVID era.

This article was previously presented as a meeting abstract at the 2022 American Roentgen Ray Society (ARRS) Annual Meeting on May 1-5, 2022.

## Materials and methods

Study design and data collection

This is a retrospective observational study that was approved by our local institutional review board and performed at a single tertiary care medical center in a large metropolitan area. The study was done from December 2018 to July 2021, a total of 32 months. We have divided pre-COVID-19 era lung V/Q scintigraphy (December 2018 to March 2020, 16 months) into lung perfusion scintigraphy during the post-COVID-19 era (April 2020 to July 2021, 16 months). A total of 878 patients were enrolled in the study. Of 868 patients, in the pre-COVID-19 era, there were 511 patients, and 357 patients in the post-COVID-19 era. In the pre-COVID-19 era and post-COVID-19 era, we determined the pretest probability of patients using Wells’ criteria [7]. Wells' criteria are clinical prediction rules used to estimate the probability of PE in patients. It assigns points based on specific clinical features: 3 points each for clinical symptoms of deep vein thrombosis (leg swelling and pain) and if other diagnoses are less likely than PE; 1.5 points each for tachycardia (heart rate > 100 beats per minute), a previous history of DVT or PE, and immobilization for more than three days or recent surgery within the past four weeks. Additionally, 1 point each is assigned for the presence of malignancy and hemoptysis. Based on the total score, patients are stratified into different categories of PE probability: high probability (> 6 points), moderate probability (2-6 points), and low probability (< 2 points) [7]. Patients with suspected PE with intermediate pretest probability and positive D-dimer test or high pretest probability who underwent lung ventilation and perfusion scintigraphy were included in the study. Ten patients who underwent lung perfusion for quantification were excluded from the study. In the pre-COVID-19 era, 511 patients and 357 patients in the post-COVID-19 era were included in the study. The collected data included the patient's demographic features (age, gender, presenting complaints, comorbidities, physical examination, and laboratory results). D-dimer level, CTPA, and extremities duplex findings were documented according to availability. D-dimer level assay in our medical center was measured in milligrams per liter (mg/L) using an enzyme-linked immunosorbent assay (D-dimer level is considered elevated in our laboratory when it is more than 0.52 mg/L). During post-COVID-19, patients who underwent either rapid antigen or reverse transcription-polymerase chain reaction (RT-PCR) COVID-19 testing, their results were also documented in the study.

Lung V/Q scintigraphy technique

During the pre-COVID-19 era, lung V/Q scans were performed. A lung V/Q scan consists of two parts: ventilation (V) and perfusion (Q). In our institute, we use 133Xe gas (Xenon 133, 5.27-day isotope half-life, biologic half-life of 30 seconds, 81 keV photon energy) as a ventilation agent and 99mTc-MAA (Technetium 99m macro aggregated albumin (MAA), six hours isotope half-life, 140 keV photon energy) as a perfusion agent.

A facemask or mouthpiece was connected to the xenon delivery system. 133Xe was inhaled as a mixture of air, and the inhalation dose was between 5 and 20 mCi (158-740 MBq). The patient took a single breath end-inspiratory capacity first and then held it for 20 seconds; during that time, a single breath image was obtained. After that equilibrium images or wash-in images (two to three minutes) were obtained, while the patient re-breaths, the mixture of air and 133Xe in a closed system. At the end of the ventilation phase, washout images were obtained (two to three minutes). All images were performed in only one projection due to a shorter biological half-life of 133Xe. 

99mTc MAA was used as the perfusion agent in the V/Q scan. A 1-4 mCi (37-148 MBq) of 99mTc MAA was slowly injected intravenously over four to five respiratory cycles in the supine position to minimize the vertical perfusion gradient and decrease diaphragmatic motion. A total of 200,000-500,000 particles were injected. Images were performed in eight different projections (anterior, posterior, right lateral, left lateral, right anterior oblique, right posterior oblique, left anterior oblique, and left posterior oblique).

Lung perfusion (Q) scintigraphy technique

During the post-COVID-19 era, only lung perfusion (Q) imaging was performed, owing to concern about the transmissibility of COVID-19. The lung perfusion scintigraphy technique was the same as the perfusion part of the study we did in the pre-COVID-19 era.

Interpretation

The perfusion defects are wedge-shaped peripheral and segmental in distribution. According to the size of the perfusion defect, it was categorized as large (> 75% of a segment involvement), moderate (25%-75% of a segment involvement), or small (< 25% of a segment involvement) size defects. We correlated the lung perfusion scintigraphy with the conventional radiograph performed within 24 hours of the perfusion scan as a surrogate of lung ventilation. For planar V/Q imaging during the pre-COVID-19 era, interpretation was based on the modified Prospective Investigation of Pulmonary Embolism Diagnosis II (PIOPED II) (mPIOPED II) criteria [8].

The mPIOPED II criteria are used to assess the likelihood of PE based on V/Q scan results. The criteria classify findings into three primary categories: high probability (PE present), normal perfusion or very low probability (PE absent), and low or intermediate probability (non-diagnostic) [8].

High Probability (PE Present)

This category includes findings where there are two or more large segments of V/Q mismatch. One large segmental perfusion defect is considered equivalent to two moderate-sized segmental perfusion defects.

Normal Perfusion or Very Low Probability (PE Absent)

This category includes several specific conditions. Non-segmental perfusion abnormalities such as an elevated hemidiaphragm, cardiomegaly, enlarged hila, costophrenic angle effusion, and linear atelectasis without any other perfusion defects in either lung are included. It also includes one to three small segmental perfusion defects involving less than 25% of a segment, perfusion defects smaller than the corresponding radiographic lesion, a single triple matched defect in the mid or upper zone of the lung confined to one segment (matched V/Q defect with associated matching opacity on the chest radiograph), two or more matched V/Q defects with a normal chest radiograph and areas of normal perfusion elsewhere in the lungs, and pleural effusion occupying one-third or more of the pleural cavity with no other perfusion defect. Additionally, the stripe sign, which is a stripe of perfused lung tissue between a perfusion defect and the adjacent pleural surface, best seen on a tangential view, is included in this category.

Low or Intermediate Probability (Non-diagnostic)

This category includes any findings that do not fall into the high probability or normal/very low probability categories.

During the post-COVID-19 era, a ventilation scan was not performed, and Q scan interpretation was done using the perfusion-only mPIOPED II (pPIOPED II) criteria [8].

The perfusion-only mPIOPED II (pPIOPED II) criteria are used to evaluate the likelihood of PE based on lung perfusion scans, particularly in conjunction with chest radiographs (CXR). These criteria categorize findings into three main groups: high probability (PE present), normal perfusion or very low probability (PE absent), and low or intermediate probability (non-diagnostic) [8].

High Probability (PE Present) 

This category includes findings of two or more large segments of Q mismatch. A single large segmental perfusion defect is equivalent to two moderate-sized segmental perfusion defects.

Normal Perfusion or Very Low Probability (PE Absent) 

This category encompasses several specific conditions. Non-segmental perfusion abnormalities such as an elevated hemidiaphragm, cardiomegaly, enlarged hila, costophrenic angle effusion, and linear atelectasis without any other perfusion defects in either lung are included. Additionally, it includes one to three small segmental perfusion defects involving less than 25% of a segment, perfusion defects smaller than the corresponding radiographic lesion, a solitary matched (Q) defect involving less than one segment in the mid or upper lung, two or more matched Q defects with a normal chest radiograph and areas of normal perfusion elsewhere in the lungs, pleural effusion occupying one-third or more of the pleural cavity with no other perfusion defect, and the stripe sign (a stripe of perfused lung tissue between a perfusion defect and the adjacent pleural surface).

Low or Intermediate Probability (Non-diagnostic)

This category includes all other findings that do not fit into the high probability or normal/very low probability categories.

Data analysis

We relied on the independent interpretation of lung V/Q scintigraphy and lung perfusion (Q) scintigraphy by two dual-board-certified nuclear medicine physicians and a radiologist. Statistical analyses were performed using the Statistical Product and Service Solutions (SPSS, version 18; IBM SPSS Statistics for Windows, Armonk, NY) software. Descriptive statistics, including percentage (%), were used to determine the proportion of patient falls in the high, intermediate, or low probability PE scan. All P values were two-sided, considering values < 0.05 as statistically significant.

## Results

A total of 878 patients were enrolled in the study. Ten patients who underwent lung perfusion for quantification were excluded from the study. Of 868 patients, in the pre-COVID-19 era, there were 511 patients, and 357 patients in the post-COVID-19 era. In the pre-COVID-19 era, 511 patients (181 men: 330 women; mean age: 58.3 years; age range: 17-103 years) and 357 patients (122 men: 235 women; mean age: 61.5 years; age range: 6-95 years) in the post-COVID-19 era were included in the study.

**Figure 1:**
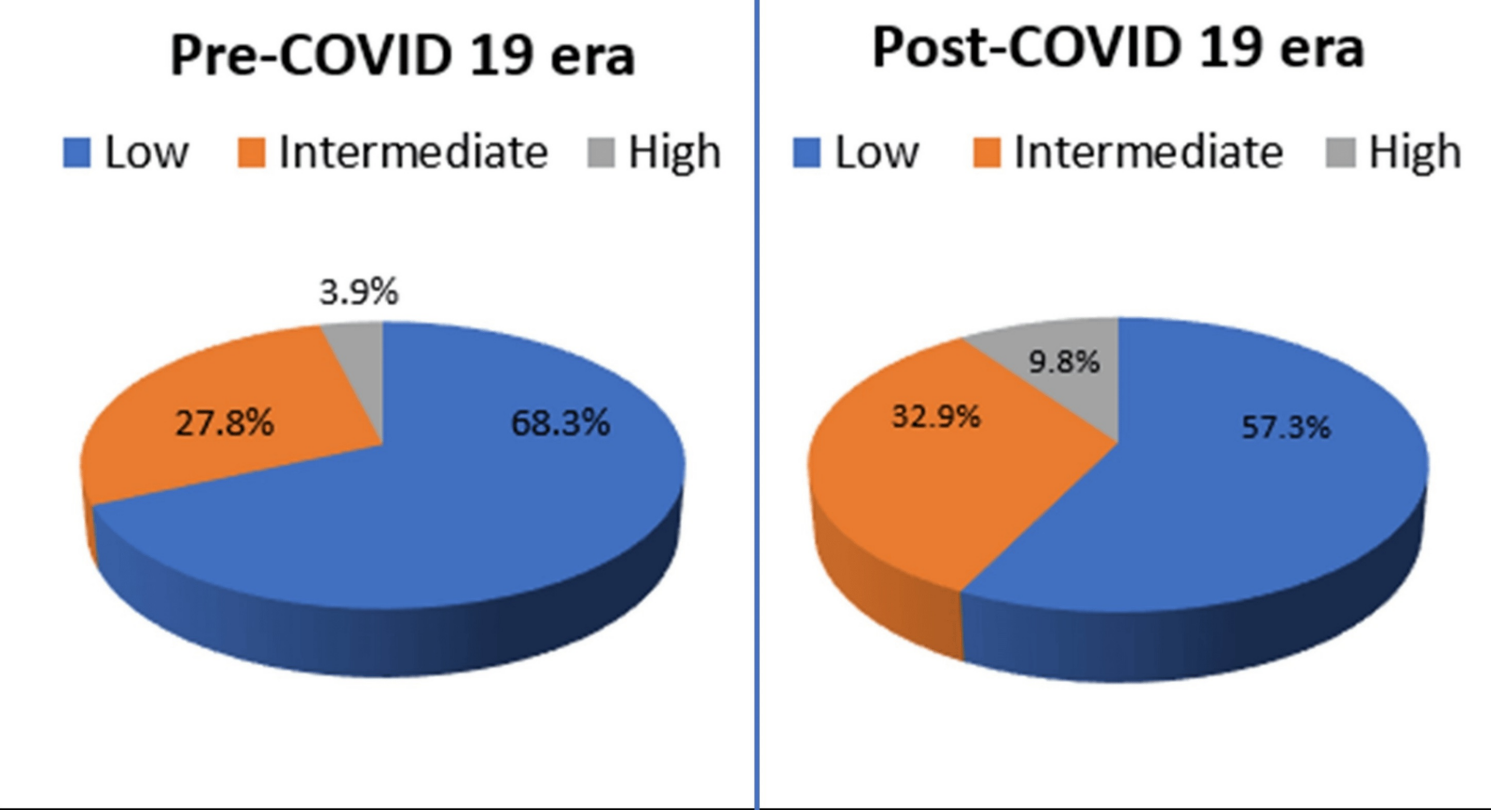
Comparison of probability scans for pulmonary thromboembolism on lung perfusion or V/Q scan in the pre-COVID-19 era to the current COVID-19 era Out of 511 patients who underwent a V/Q scan during the pre-COVID-19 era, 68.3% had low, 27.8% had intermediate, and 3.9% had a high probability scan for pulmonary thromboembolism. Out of 347 patients who underwent lung perfusion scintigraphy during the post-COVID-19 era, 57.3% had low, 32.9% had indeterminate or nondiagnostic scans, and 9.8% had high probability scans for pulmonary thromboembolism.

As expected, there was approximately a 30% decline (154 fewer lung scans) in the overall number of lung scans performed during the post-COVID-19 era pandemic (357 patients), as compared to the pre-COVID-19 era where 511 V/Q scans were performed. Out of 511 patients who underwent a V/Q scan during the pre-COVID-19 era, 349 (68.3%) had low, 142 (27.8%) had intermediate, and 20 (3.9%) had high probability scan for pulmonary thromboembolism. Out of 347 patients who underwent lung perfusion scintigraphy during the post-COVID-19 era, 199 (57.3%) had low, 114 (32.9%) had indeterminate or nondiagnostic scans, and 34 (9.8%) had high probability scans (Figure 1) for pulmonary thromboembolism(Figure 1 and Table 1).

**Table 1:** Probability of pulmonary thromboembolism on the V/Q scan during the pre-COVID-19 era to the lung perfusion scintigraphy during the post-COVID-19 era

Probability of pulmonary thromboembolism	V/Q scan during the pre-COVID19 era, number (percentage in %)	Lung perfusion scintigraphy during the post-COVID19 era Number (percentage in %)
Low probability	349 (68.3 %)	199 (57.3 %)
Intermediate probability	142 (27.8 %)	114 (32.9 %)
High probability	20 (3.9 %)	34 (9.8 %)

**Figure 2:**
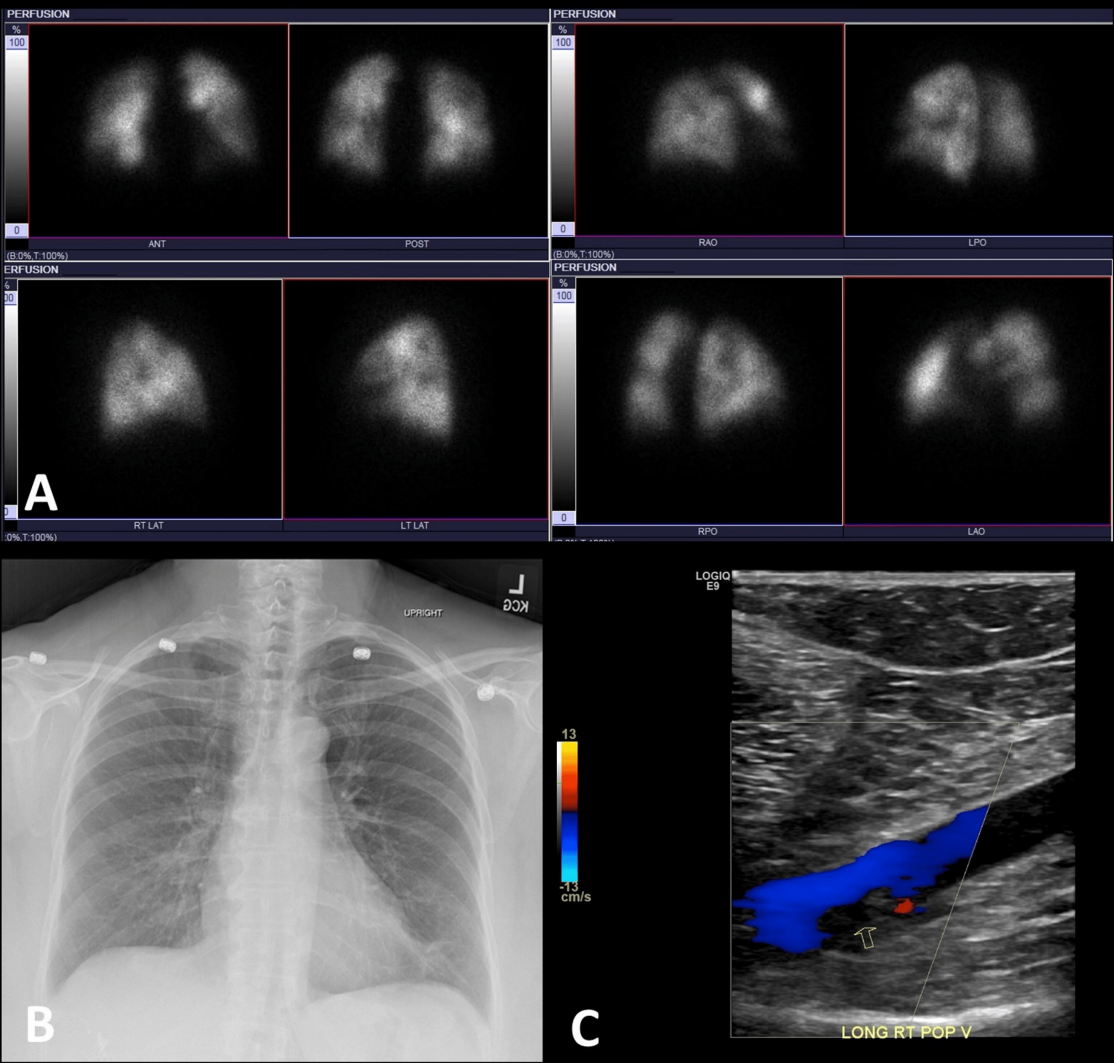
High probability for PE in a 66-year-old woman positive with COVID-19 (A) Images from lung perfusion scintigraphy performed with 99mTc-MAA, large-sized segmental perfusion defects in the posterior segment of the right upper lobe and inferior lingula. Moderate-sized segmental perfusion defects in the anteromedial basal segment of the left lower lobe and anterior basal segment of the right lower lobe. Based on modified PIOPED II perfusion-only criteria, the study is best classified as a high-probability scan for pulmonary embolism. (B) A chest radiograph is normal. (C) Color duplex study shows partially occlusive acute deep venous thrombosis within the right popliteal vein (yellow open arrow).

**Figure 3:**
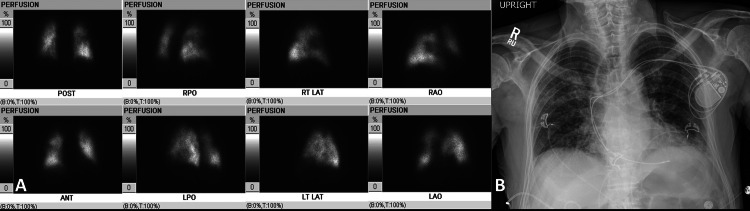
High probability for PE in an 85-year-old male positive with COVID-19 (A) Images from lung perfusion scintigraphy performed with 99mTc-MAA, mild heterogeneous distribution of tracer activity in bilateral lungs. Large-sized perfusion defect in the anterior segment of the right upper lobe. Two moderate-sized perfusion defects in the posterior segment of the right upper lobe, as well as the apicoposterior segment of the left upper lobe. Based on the modified PIOPED II perfusion-only criteria, the study is best classified as a high-probability scan for pulmonary embolism. (B) A chest radiograph shows no radiographic evidence of the acute cardiopulmonary process. Right upper and bibasilar ground-glass and reticular opacities related to known interstitial lung disease. Left chest cardiac device with two intact intracardiac leads projecting over the right atrium and right ventricle. External EKG leads.

**Figure 4:**
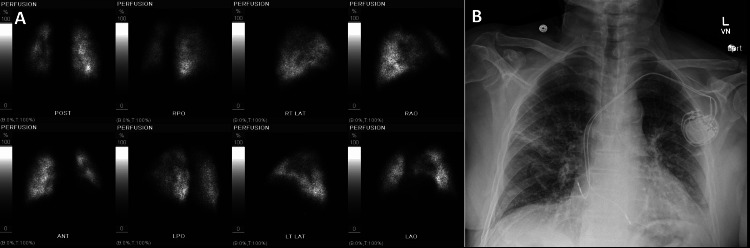
Intermediate probability for PE in a 70-year-old male positive with COVID-19 (A) Images from lung perfusion scintigraphy performed with 99mTc-MAA, heterogeneous tracer uptake within the bilateral lung parenchyma. Moderate-sized segmental perfusion defect in the superior segment of the left lower lobe, better visualized left anterior and posterior images. Based on the modified PIOPED II perfusion-only criteria, the study is best classified as an intermediate probability scan for pulmonary embolism. (B) A chest radiograph shows scattered patchy ground-glass opacities visualized in the right upper and mid lung, as well as the left perihilar region, likely related to known COVID-19 infection. Stable left-sided cardiac device with intact leads projecting over the right atrium and right ventricle.

**Figure 5:**
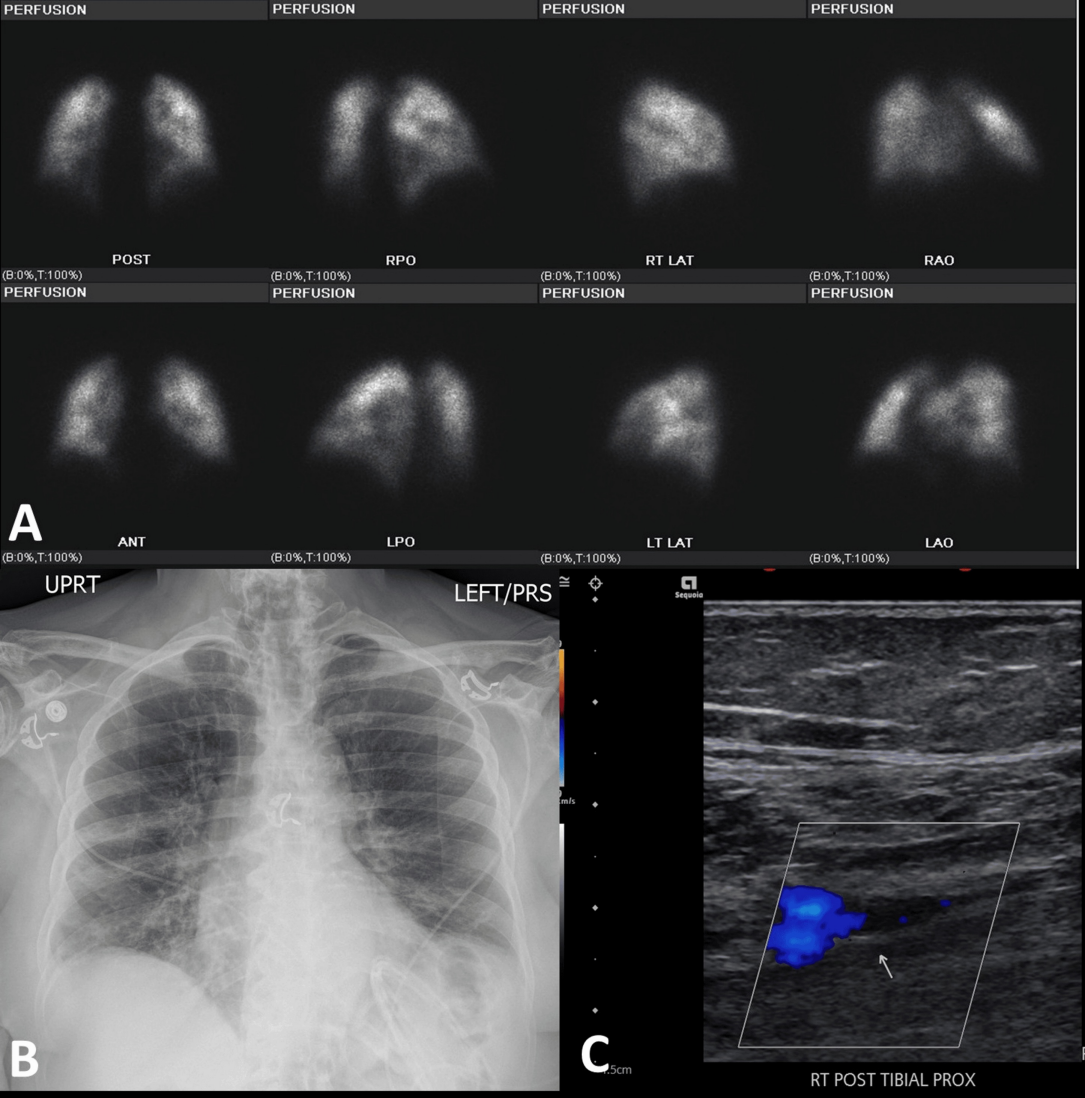
Intermediate probability for PE in a 71-year-old woman positive with COVID-19 (A) Images from lung perfusion scintigraphy performed with 99mTc-MAA, heterogeneous distribution of tracer activity in bilateral lungs, with scattered multiple perfusion defects, for example, in the superior segments of the bilateral lower lung lobes and the perihilar regions. Based on the modified PIOPED II perfusion-only criteria, the study is best classified as an intermediate probability scan for pulmonary embolism. (B) A chest radiograph shows bilateral ground-glass airspace opacities most compatible with COVID-19 infection. Low lung volumes and left lung base atelectasis. (C) A color duplex study shows acute partially occlusive deep vein thrombosis in one of the paired right posterior tibial veins (white arrow).

**Figure 6:**
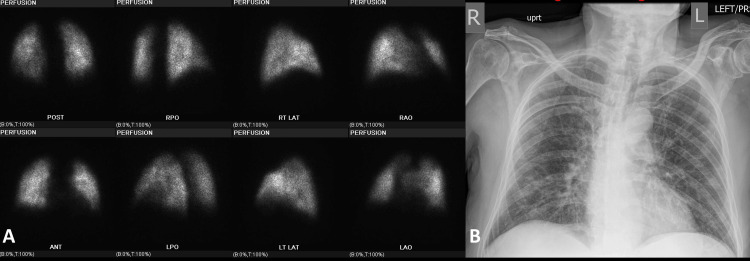
Low probability for PE in a 76-year-old male positive with COVID-19 (A) Images from lung perfusion scintigraphy performed with 99mTc-MAA, near homogeneous tracer distribution in both lungs. No distinct segmental perfusion defect noted. Based on the modified PIOPED II perfusion-only criteria, the study is best classified as a low-probability scan for pulmonary embolism. (B) A chest radiograph shows patchy ground-glass opacities of both lungs in a peripheral distribution in a known case of COVID-19.

Representative cases of low-, intermediate-, and high-probability scans are shown in Figures 2-6.

Out of 347 patients in post-COVID-19 eras, 287 underwent either rapid antigen or RT-PCR COVID-19 testing. Forty-five were COVID-19 positive, and 243 were COVID-19 negative. Of 243 COVID-19-negative patients, 143 (58.9%) had low, 75 (30.9%) had intermediate, and 25 (10.2%) had high probability scans. Of 45 documented COVID-19-positive patients, 18 (40%) had low, 22 (48.9%) had intermediate, and five (11.1%) had high-probability scans. Out of the 187 patients who underwent duplex ultrasound study of extremities in the post-COVID-19 era, 37 (19.8%) patients were positive for deep vein thrombosis. In COVID-19-positive patients, five (15.63%) patients were positive for deep vein thrombosis on duplex ultrasound (Figures 2C, 5C). Out of the 36 patients who underwent a CTPA study for PE in the post-COVID-19 era, eight (22.2%) patients were positive for PE on CTPA. In COVID-19-positive patients, one (25%) patient was positive for PE on CTPA. Total of 212 patients who underwent D-dimer analysis during the post-COVID-19 era, 18 (8.50%) patients had a normal level of serum D-dimer, and 194 (91.50%) patients had elevated levels (> 0.52 mg/L) of serum D-dimer level. In COVID-19-positive patients, 40 (97.57%) patients had elevated serum D-dimer levels. The average D-dimer level is 3.83 mg/L in patients with elevated D-dimer levels.

## Discussion

Lung V/Q scintigraphy is the oldest noninvasive diagnostic study for the evaluation of PE. It was first used in humans in 1964 by Quinn et al. [2]. PE was first reported on CT in 1978, till the time that the V/Q scan was the only imaging modality for PE [2,3]. The CTPA study is the routinely used main investigation to rule out pulmonary thromboembolism. However, in the event that a patient is allergic to iodinated contrast and a CT scan cannot be performed, a V/Q scan is an alternative. An additional indication to perform a V/Q scan is when the CT scan is inconclusive due to artifacts. Coronavirus disease 2019, also called COVID-19, is a highly contagious disease caused by severe acute respiratory syndrome coronavirus 2 (SARS-CoV-2). Coronaviruses are positive-stranded RNA viruses with spikes of glycoproteins on the envelope that give a crown-like appearance [4-6]. Exposure to respiratory droplets containing the infectious virus from close contact, with presymptomatic, asymptomatic, or symptomatic individuals acts as the primary mode of transmission of SARS-CoV-2. The virus can be viable on plastic and stainless steel for up to two to three days; this seems to be a higher nosocomial transmission risk associated with SARS-CoV-2 [5]. The current guideline from the CDC recommends that healthcare personnel while performing aerosol-generating procedures should optimally wear an N95 or higher-level respirator, gloves, gown, and eye protection [4-6]. COVID-19 is associated with an increased risk of PE due to factors such as hypercoagulability, endothelial damage, prolonged immobilization, and severe inflammatory response. These conditions promote clot formation, increasing the likelihood of PE in affected individuals [9].

The typical protocol for the V/Q scan, about the ventilation phase of the scan, carries a risk of radioactive contamination due to leakage of the aerosol from the closed delivery system into the room [10]. Symptomatic patients with COVID-19 are prone to cough, and this may further increase exposure of the nuclear medicine staff workers to aerosolized secretion, especially after inhalation of radiopharmaceuticals [6]. The Society of Nuclear Medicine and Molecular Imaging (SNMMI) released a statement on March 19, 2020, concerning V/Q lung scans, specifically the risk of spread of COVID-19 to patients and staff related to the ventilation portion of the study. Additionally, many institutes had opted out ventilation portion of the study and implemented a lung perfusion-only study [11]. Our institute also followed the same.

During the pre-COVID-19 era, the interpretation of planar V/Q imaging was based on the modified PIOPED II (mPIOPED II) criteria [12]. According to Sostman et al., mPIOPED II has 77% sensitivity for the PE-present category and a specificity of 98% for the PE-absent category [13].

During the post-COVID-19 era, a ventilation scan was not performed, and Q scan interpretation was done using the perfusion-only modified PIOPED II (pPIOPED II) criteria [14]. The size and shape of the defect are important in categorizing large (> 75% of a segment involvement), moderate (25%-75% of a segment involvement), or small (< 25% of a segment involvement) size defects [15].

According to a study by Freudenberg et al. on the global impact of COVID-19 on nuclear medicine departments, there was a 56% reduction in lung scans globally during COVID-19 [16]. In our study also, there was a 30% decline in the overall number of lung scans during the post-COVID-19 eras. According to a study done by Calve-Romeo et al., 10.5% intermediate-probability scans and 28.8% high-probability V/Q scans [17]. Bocher et al. proposed that 16% of V/Q scans were high probability [18]. In our study in the pre-COVID-19 era, there were 27.8% intermediate-probability scans and 3.9% high-probability scans. The incidence of intermediate- and high-probability scans was 4.7% in a study done by Gayed et al. during the COVID-19 pandemic [19]. However, in our study, the incidence of intermediate and high probability scans is 32.9% and 9.8%, respectively. This is because the lung parenchymal changes in chest radiographs increase the intermediate-probability scans. In an observational study by Tacquard et al., 500 COVID-19 patients admitted to eight intensive care units in France reported 22.7% thrombotic complications [20], and 15.63% of patients with COVID-19 demonstrated deep vein thrombosis in the vascular duplex study. Thus, hypercoagulability and vascular complications associated with COVID-19 increase the high probability of scans.

Lung perfusion scintigraphy combined with chest radiography can provide diagnostic accuracy similar to both V/Q scan and CTPA, at lower radiation dose and cost.

Some centers are doing lung perfusion scans with SPECT imaging and low-dose CT, instead of the correlation with conventional radiographs. The advantages of doing SPECT/CT are higher diagnostic accuracy and simultaneous diagnosis of lung parenchymal or pleural disease. The disadvantage of doing SPECT/CT is additional radiation to the patient.

The limitations of our study are the retrospective in nature, fewer variables, and lack of assessment of observer bias. COVID-19 is associated with cardiac complications, which alter the interpretation of the perfusion part of the scan.

As these artificial intelligence (AI) systems continue to evolve and become more advanced, they hold the potential to transform various industries and greatly enhance the quality of life for people around the world [21]. The AI algorithm demonstrated high sensitivity, specificity, and accuracy for detecting PE on CECT scans in COVID-19 patients, though its accuracy was affected by the mean attenuation of the pulmonary vasculature, raising concerns about the legitimacy of the binary outcomes [22]. More dedicated research is needed in the development of AI for lung perfusion.

## Conclusions

The perfusion part of the lung scan is sufficient for the evaluation of acute PE in the majority of the patients. Eliminating the ventilation part of the V/Q scan had only a minimal impact on the interpretation of the study. A 5.1% rise in indeterminate-probability and non-diagnostic scans using the perfusion-only modified PIOPED II criteria during the post-COVID-19 era is thought to be due to underlying lung parenchymal involvement seen on chest radiographs of the majority of COVID-19 patients. Another 5.9% rise in high-probability scans as compared to the pre-COVID-19 era is due to known hypercoagulability and vascular complications in COVID-19 patients.
